# A Filthy Atmosphere

**Published:** 1877-09

**Authors:** 


### A FILTHY ATMOSPHERE.

We were passing a corner on two prominent streets the other evening, where there were assembled perhaps a dozen ladies and gentlemen waiting for the cars, when our nostrils were assailed with the most fearful stench of an atmosphere, to be found anywhere outside of a class-room in one of our public schools. Looking around for the cause, we found it to be the air still adhering to the clothing of these people who had just come from one of the negro-minstrel shops, where they had been spending the evening; and although they had walked three or four blocks in the open air, the stench stuck to them yet!

Reader, if you are well and would keep so, never visit these ill-ventilated holes; if you are an invalid, and go there, you are hurrying yourself out of the world at railroad pace.

